# Supplementary material for: A Novel LC System Embeds Analytes in Pre-formed Gradients for Rapid, Ultra-robust Proteomics
Source: Mol Cell Proteomics. 2018 Aug 13;17(11):2284–96. doi: 10.1074/mcp.TIR118.000853 (PMC6210218; doi:10.1074/mcp.TIR118.000853)
Supplement: supplemental Fig. S1A, S1B [file TIR118.000853_index.html]

Supplement to A novel LC system embeds analytes in pre-formed gradients for rapid, ultra-robust proteomics | Molecular & Cellular Proteomics

## Supplemental Data

- Supplemental figures - Supplemental figures
- Supplemental tables - Supplemental tables
