# Supplementary material for: A Novel LC System Embeds Analytes in Pre-formed Gradients for Rapid, Ultra-robust Proteomics
Source: Mol Cell Proteomics. 2018 Aug 13;17(11):2284–96. doi: 10.1074/mcp.TIR118.000853 (PMC6210218; doi:10.1074/mcp.TIR118.000853)
Supplement: supplemental Fig. S1A, S1B [file 138104_1_supp_168371_pbt87m.pdf]

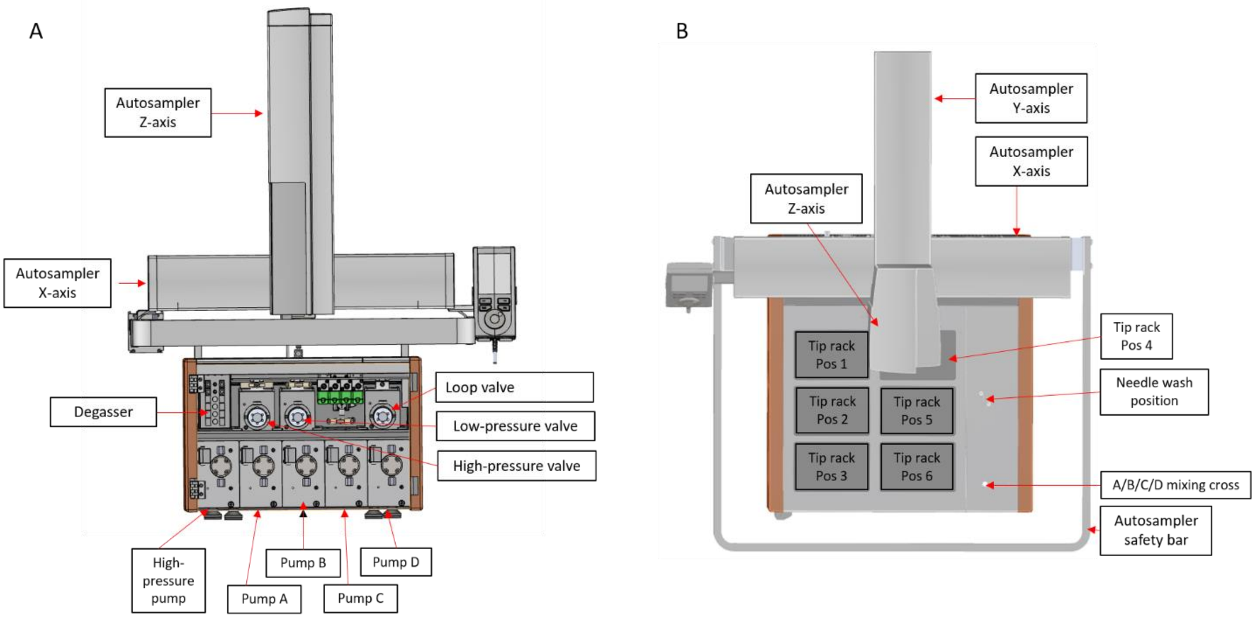

Supplemental Fig. S1: Overview of the Evosep One system. *A,* Front view of the Evosep One with main the components indicated. *B,* Top view of the instruments with the robotic autosampler arms and the tip rack positions.

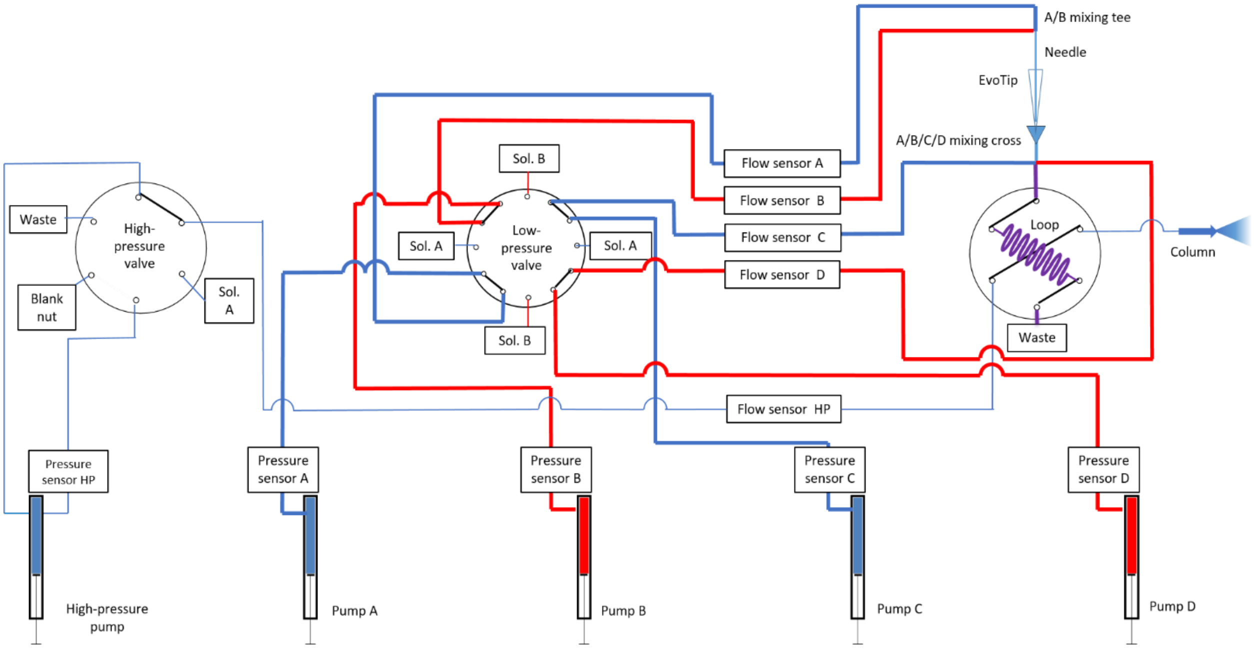

Supplemental Fig. S2: Tip elute and gradient formation. The active flow paths in this and the following figures are indicated by bold blue and bold red colors (buffer A and buffer B, respectively).

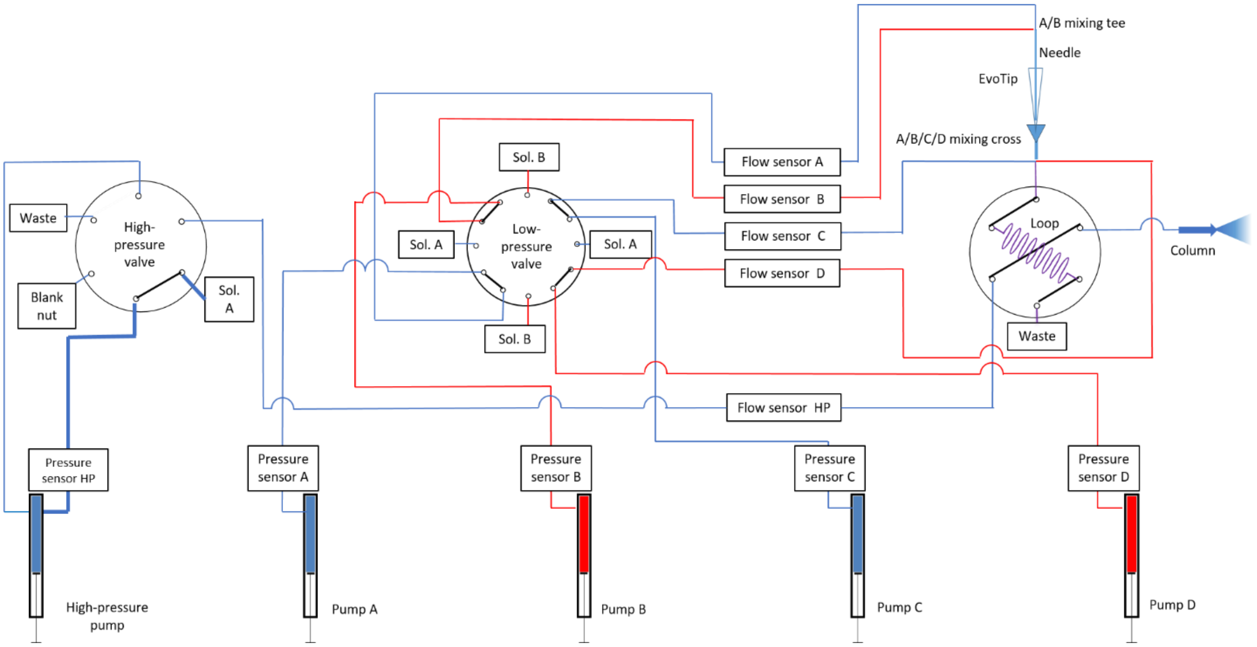

Supplemental Fig. S3: Refill high-pressure pump.

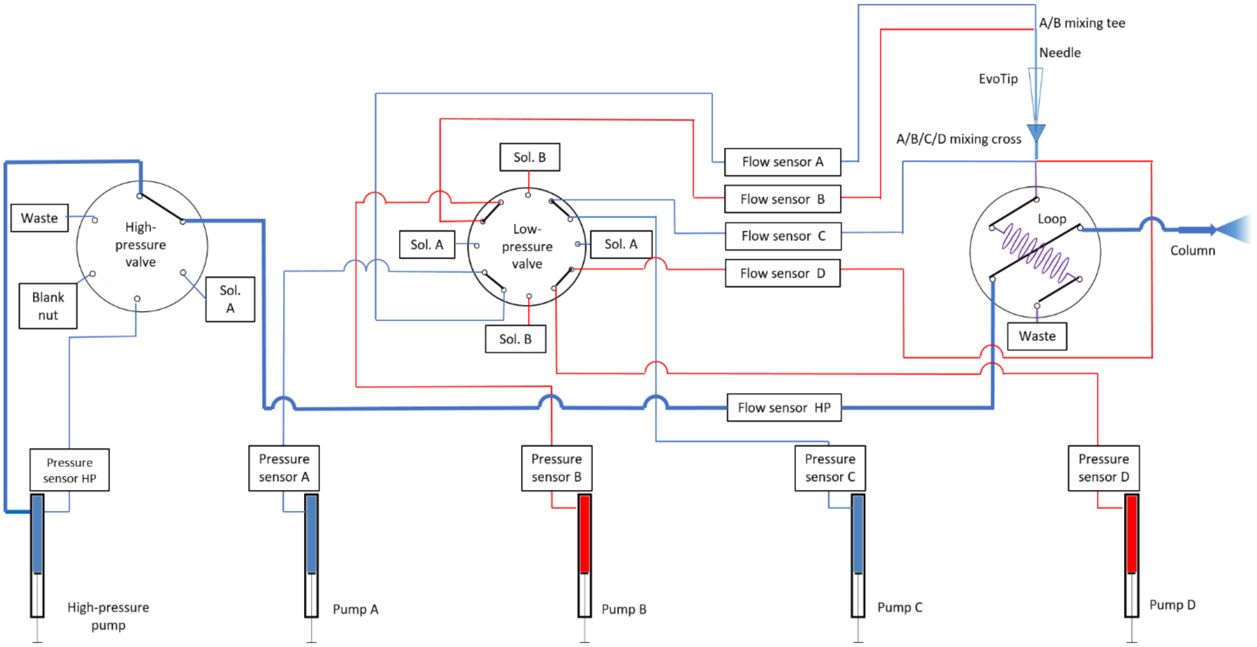

Supplemental Fig. S4: Equilibrate analytical column.

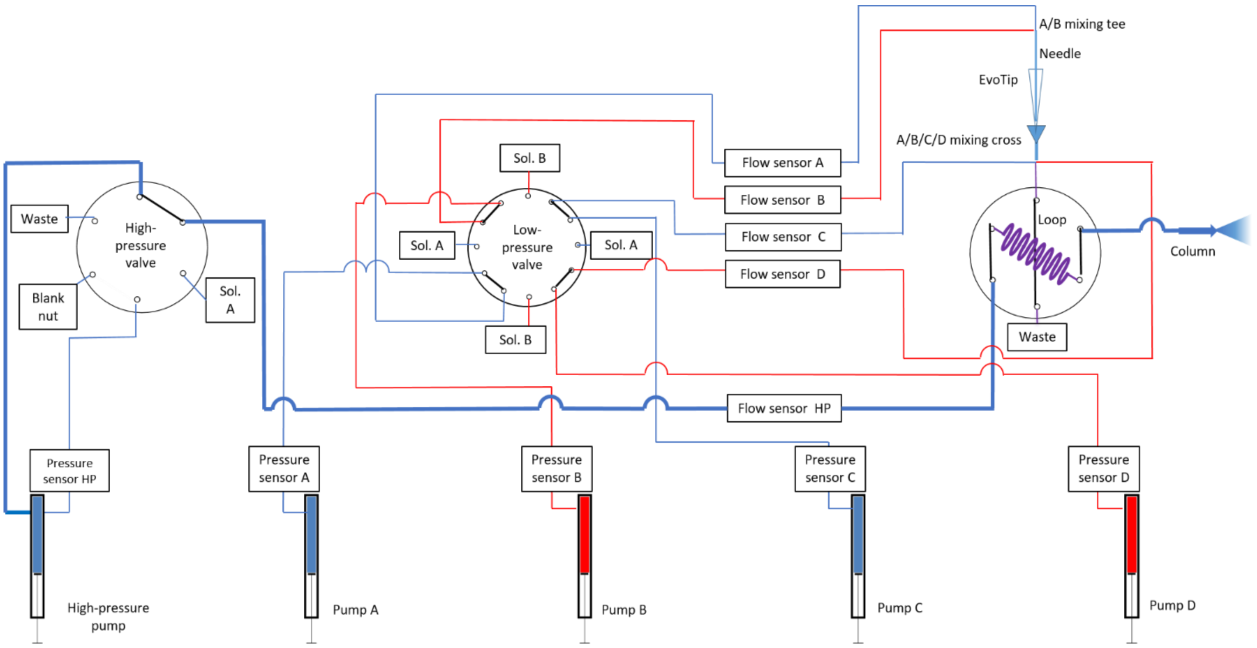

Supplemental Fig. S5: Run gradient to analytical column.

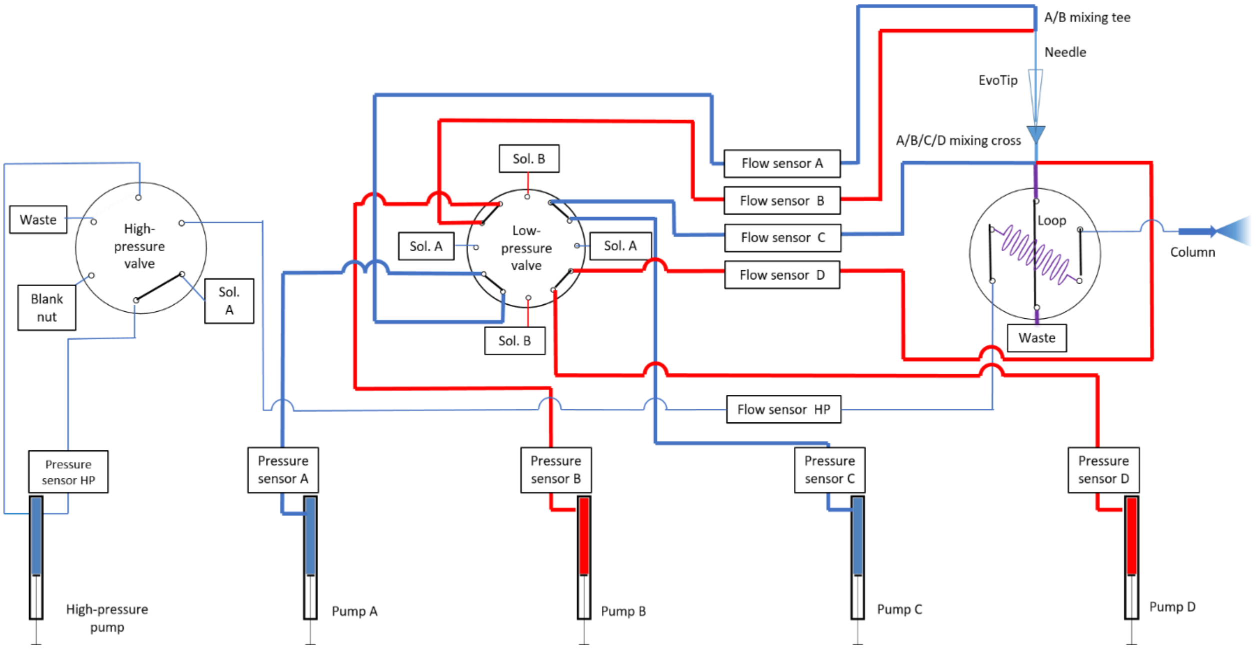

Supplemental Fig. S6: Mixing cross wash.

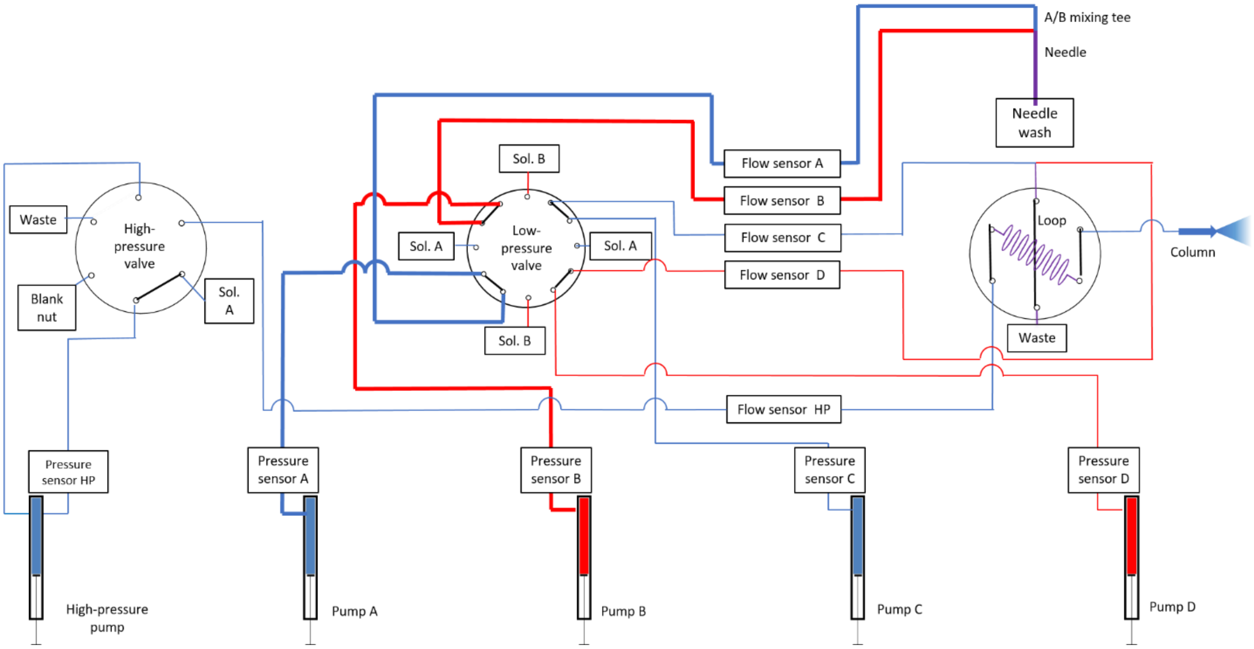

Supplemental Fig. S7: Needle wash.

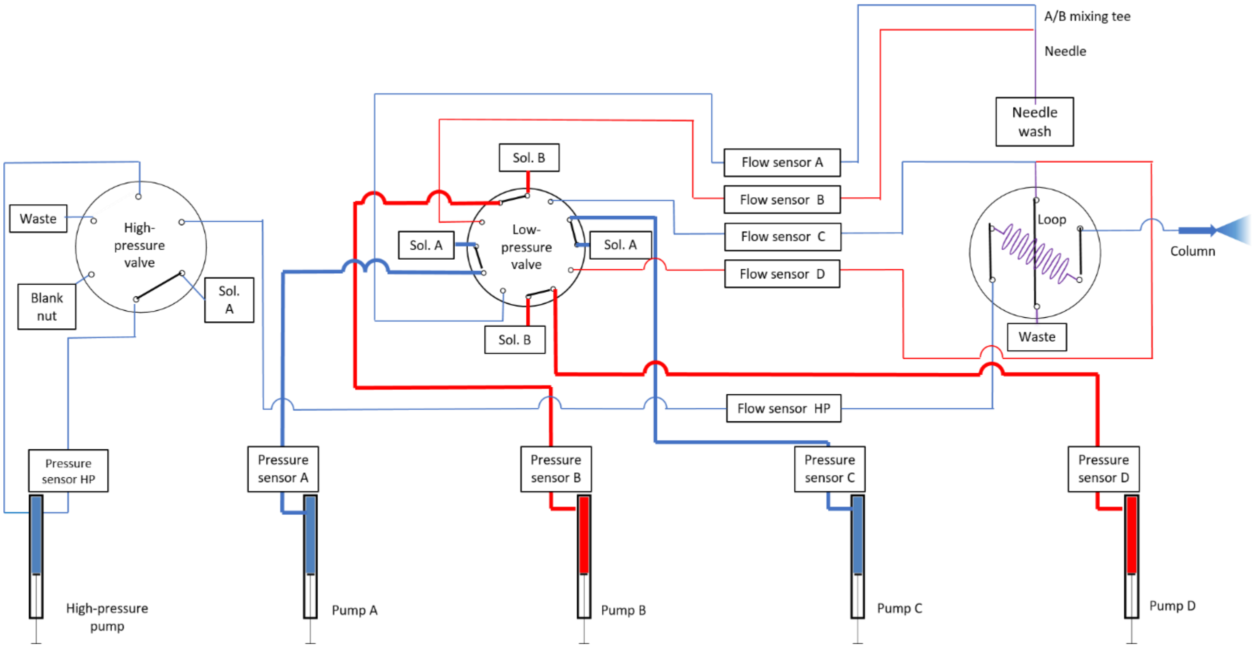

Supplemental Fig. S8: Refill pumps A/B/C/D.

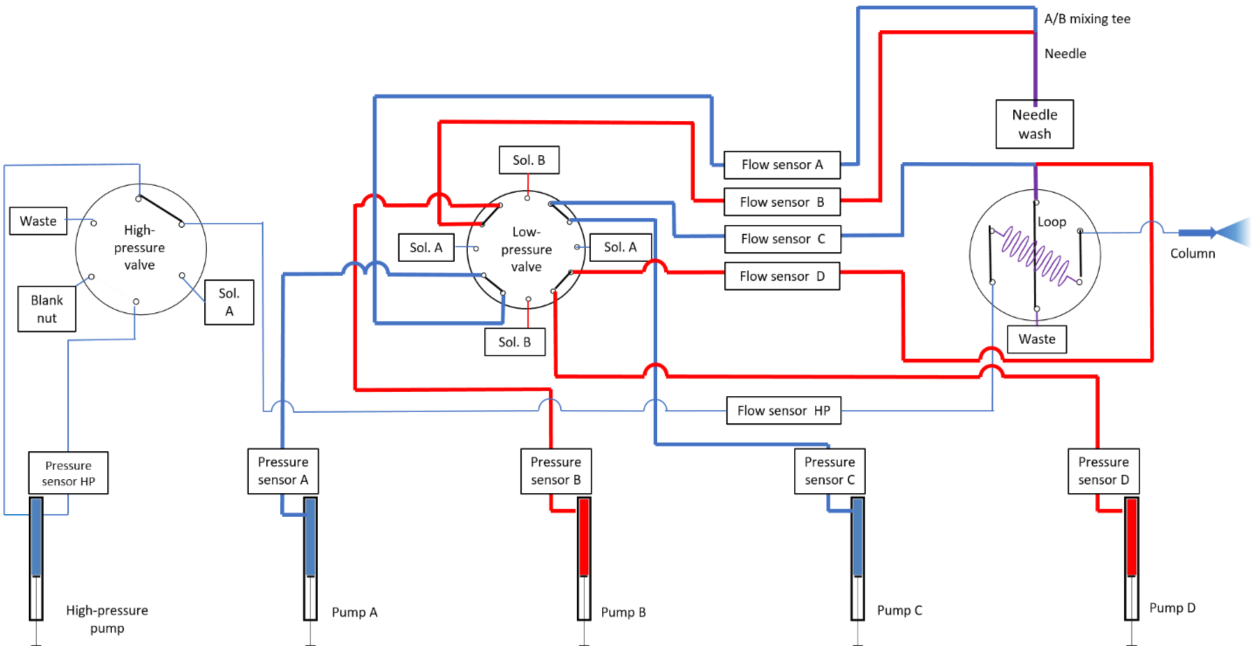

Supplemental Fig. S9: Align solvent pumps A/B/C/D.

A

Peptide	RT(min)	stdev	RT (sec)	stdev1	FWHM(s)	stdev2	TailFWHM	stdev3	Width13.4(s)	stdev4	Tail13.4	stdev5	Area	RSD
LGGGPGGGDGSR	4.5	0.1	271.1	5.6	5.8	0.1	1.3	0.2	16.2	0.6	2.5	0.2	4894793.3	0.4
LGGGPGGGDFR	10.1	0.1	608.4	7.8	19.3	0.8	1.1	0.0	46.7	3.9	2.0	0.2	184184532.8	0.2
LLGGPGGDFR	18.1	0.1	1084.6	6.4	18.7	0.5	1.2	0.2	35.1	0.3	1.4	0.1	346537162.0	0.1
DRVYIHPF	21.4	0.1	1281.0	5.1	15.5	0.5	1.4	0.3	28.3	0.3	1.6	0.3	191948552.5	0.1
E[-18]HWSYGLRPG	22.2	0.1	1329.9	4.8	14.1	0.3	1.1	0.3	25.3	0.5	1.2	0.2	350241595.0	0.1
LLLGPGDFR	27.6	0.1	1655.1	7.7	16.6	0.4	1.0	0.4	29.4	0.6	1.1	0.3	698918754.3	0.2
LLLPLDFR	35.5	0.1	2128.6	6.3	14.9	0.6	1.4	0.3	27.0	0.7	1.4	0.2	239894349.3	0.1
LLLLPLDFR	40.5	0.1	2430.9	4.9	17.0	2.4	1.1	0.3	31.9	2.6	1.3	0.1	177652157.8	0.1

B

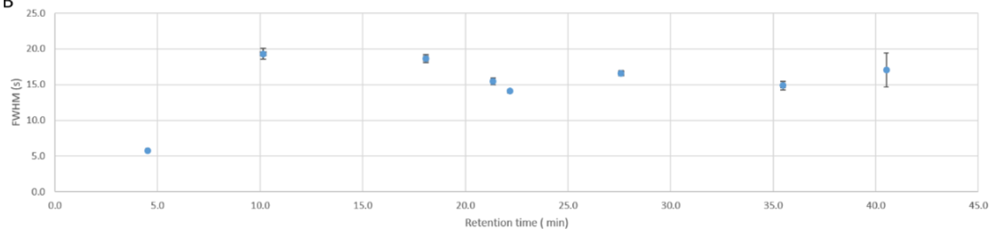

C

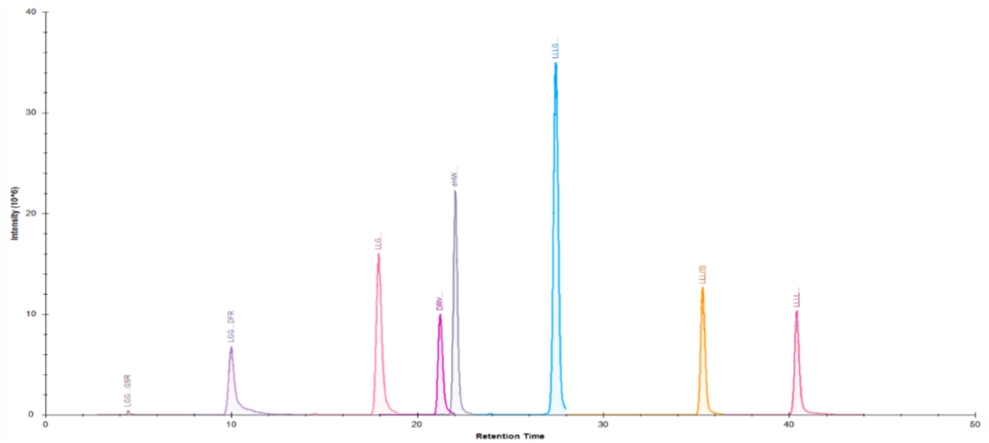

D

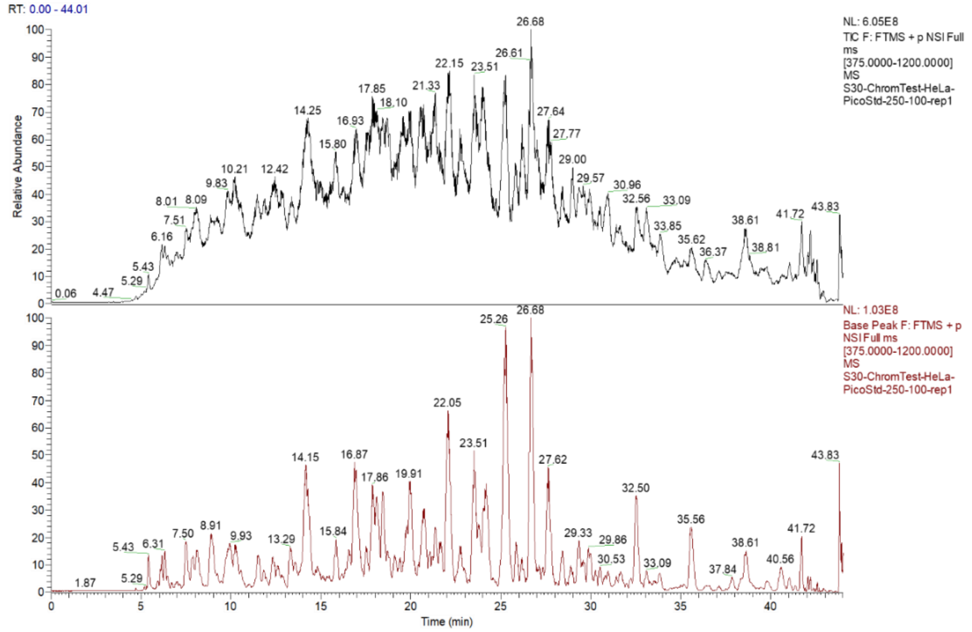

Supplemental Fig. S10: Chromatographic performance of the 30 samples per day method. *A,* Parameters describing the chromatographic performance for eight peptides of the PicoSure synthetic peptide standard. *B,* Retention time variation versus the variation in FWHM for quadruplicate measurements. *C,* Extracted chromatogram for the eight synthetic peptides. *D,* The upper panel shows the TIC and the lower panel the base peak chromatogram for the HeLa spiked in with the peptide standard.

A

Peptide	RT(min)	stdev	RT (sec)	stdev1	FWHM(s)	stdev2	TailFWHM	stdev3	Width13.4(s)	stdev4	Tail13.4	stdev5	Area	RSD
LGGGPGGGDGSR	1.97	0.02	117.98	1.37	3.17	0.30	1.28	0.36	10.20	1.88	2.62	0.71	4838574.50	0.22
LGGGPGGGDFR	4.38	0.04	262.58	2.48	7.49	0.34	1.35	0.50	18.22	1.38	1.98	0.19	137659984.50	0.09
LLGGPGGDFR	7.89	0.12	473.51	7.12	8.32	0.27	1.04	0.44	16.68	0.14	1.26	0.26	320506006.75	0.04
DRVYIHPF	9.45	0.11	567.28	6.82	6.62	0.44	1.46	0.37	12.48	0.17	1.55	0.19	158794870.00	0.08
E[-18]HWSYGLRPG	9.97	0.11	598.43	6.66	0.32	0.03	-18.97	13.81	1.16	0.53	1.92	1.59	2020610.50	0.64
LLLGPGDFR	12.61	0.07	756.40	3.98	9.81	0.66	1.24	0.38	17.69	0.69	1.07	0.18	608766305.50	0.03
LLLPLDFR	16.46	0.07	987.47	4.22	7.34	0.64	1.45	0.48	13.54	0.37	1.49	0.21	316287459.00	0.09
LLLLPLDFR	18.71	0.05	1122.40	3.17	4.48	0.11	1.05	0.26	8.07	0.32	1.13	0.17	160309390.50	0.11

B

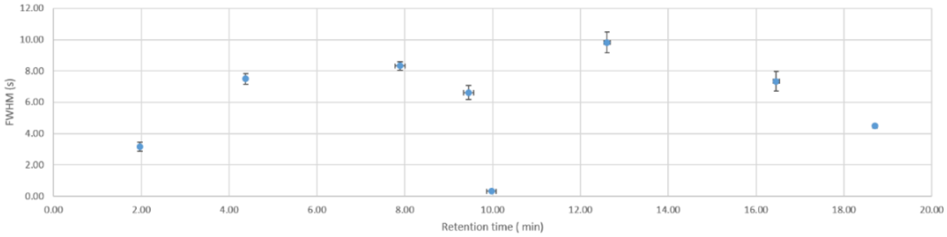

C

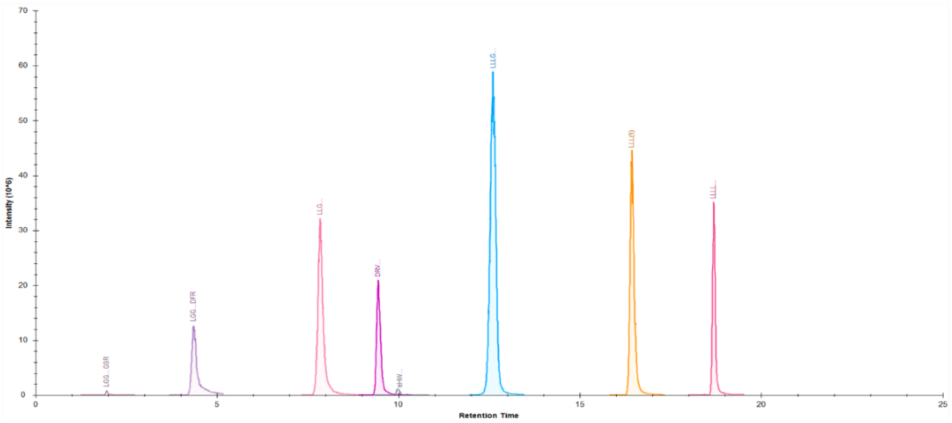

D

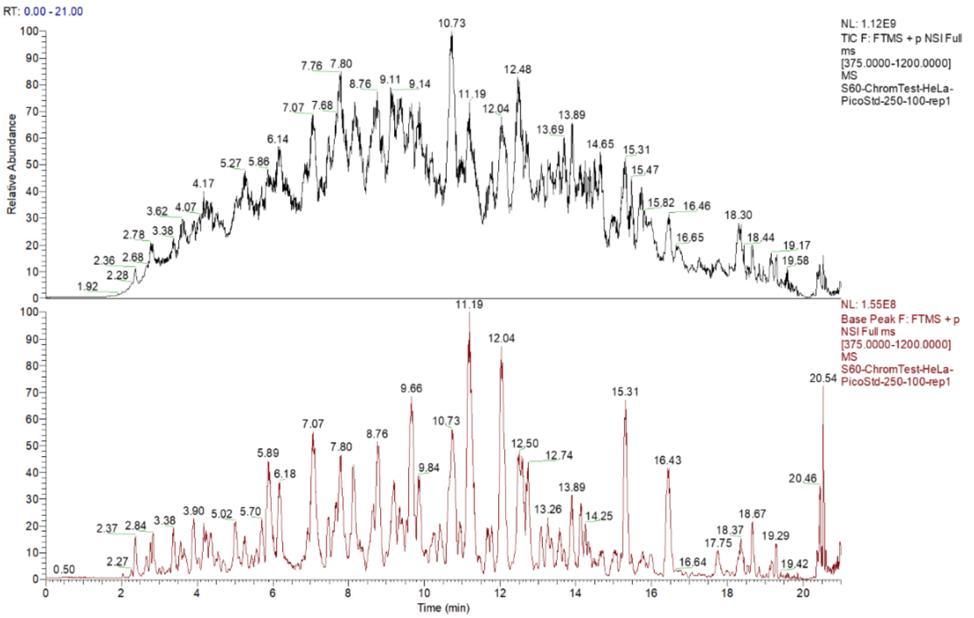

Supplemental Fig. S11: Chromatographic performance of the 60 samples per day method. *A,* Parameters describing the chromatographic performance for eight peptides of the PicoSure synthetic peptide standard. *B,* Retention time variation versus the variation in FWHM for quadruplicate measurements. *C,* Extracted chromatogram for the eight synthetic peptides. *D,* The upper panel shows the TIC and the lower panel the base peak chromatogram for the HeLa spiked in with the peptide standard.

A

Peptide	RT(min)	stdev	RT (sec)	stdev1	FWHM(s)	stdev2	TailFWHM	stdev3	Width13.4(s)	stdev4	Tail13.4	stdev5	Area	RSD
LGGGPGGGDGSR	2.26	0.03	135.50	1.53	2.36	0.19	1.16	0.09	6.13	0.60	1.83	0.08	6151902.25	0.11
LGGGPGGGDFR	3.63	0.03	217.87	1.96	4.43	0.20	0.95	0.29	9.93	0.55	1.33	0.27	96967144.75	0.11
LLGGPGGDFR	5.35	0.04	320.88	2.19	5.46	0.29	1.10	0.31	11.96	0.20	1.47	0.18	216940125.75	0.11
DRVYIHPF	6.12	0.04	367.29	2.59	4.12	0.33	1.30	0.50	7.99	0.24	1.65	0.36	118332588.50	0.05
E[-18]HWSYGLRPG	6.34	0.05	380.60	2.87	0.96	0.44	3.30	2.65	1.76	0.36	2.65	1.98	2906595.75	0.77
LLLGPGDFR	7.46	0.04	447.90	2.59	5.25	0.14	1.14	0.19	9.88	0.10	1.03	0.07	388432088.00	0.12
LLLPLDFR	9.19	0.03	551.64	1.64	3.80	0.10	1.14	0.23	6.69	0.11	1.29	0.13	162804860.50	0.17
LLLLPLDFR	9.91	0.02	594.81	1.28	2.21	0.06	1.17	0.34	4.03	0.10	1.24	0.23	125390574.75	0.14

B

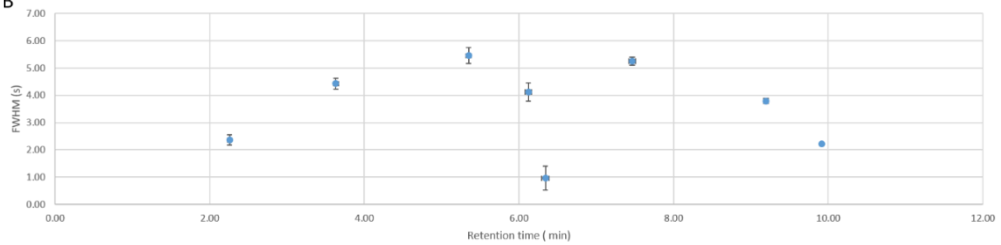

C

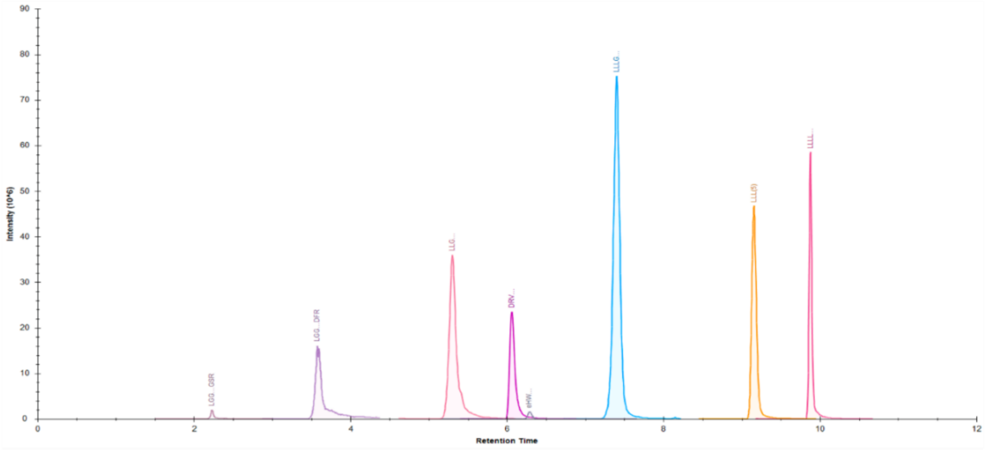

D

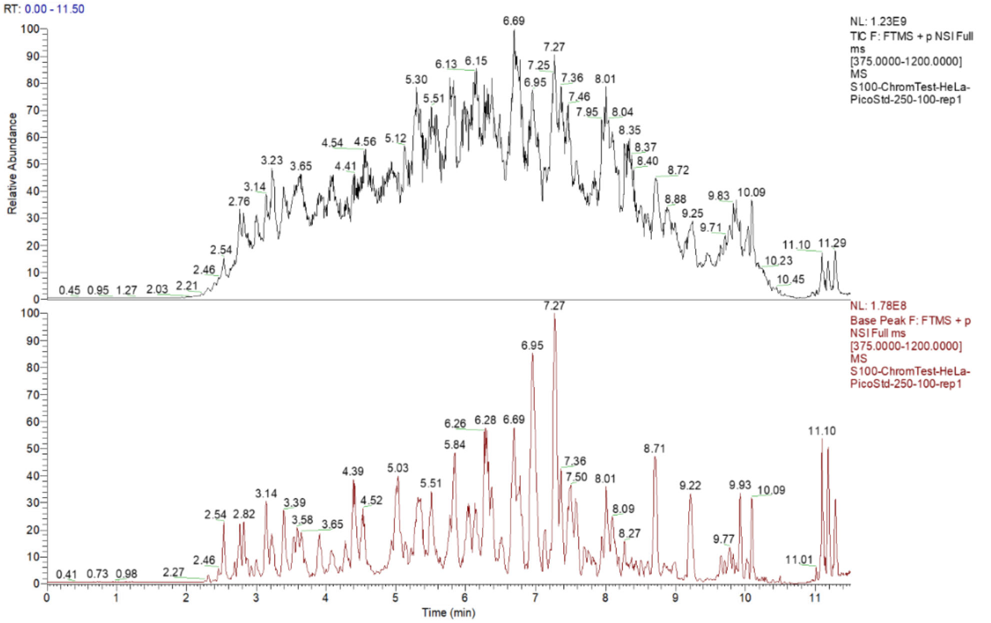

Supplemental Fig. S12: Chromatographic performance of the 100 samples per day method. *A,* Parameters describing the chromatographic performance for eight peptides of the PicoSure synthetic peptide standard. *B,* Retention time variation versus the variation in FWHM for quadruplicate measurements. *C,* Extracted chromatogram for the eight synthetic peptides. *D,* The upper panel shows the TIC and the lower panel the base peak chromatogram for the HeLa spiked in with the peptide standard.

A

Peptide	RT(min)	stdev	RT (sec)	stdev1	FWHM(s)	stdev2	TailFWHM	stdev3	Width13.4(s)	stdev4	Tail13.4	stdev5	Area	RSD
LGGGPGGGDGSR	1.27	0.01	75.93	0.65	1.53	0.13	1.29	0.07	5.09	0.40	2.64	0.12	3903547.00	0.09
LGGGPGGGDFR	1.84	0.01	110.44	0.69	1.86	0.12	1.22	0.31	4.95	0.67	1.96	0.37	42007025.50	0.12
LLGGPGGDFR	2.54	0.03	152.54	1.67	3.01	0.11	1.06	0.23	7.71	0.58	1.72	0.04	96359152.25	0.12
DRVYIHPF	2.91	0.03	174.45	1.80	2.68	0.08	1.25	0.13	6.09	0.38	2.13	0.14	72325071.75	0.12
E[-18]HWSYGLRPG	2.98	0.03	178.63	1.78	2.73	0.24	1.48	0.33	6.14	0.36	2.28	0.40	136287411.00	0.03
LLLGPGDFR	3.63	0.03	217.68	1.78	3.19	0.07	1.17	0.29	7.29	0.19	1.19	0.13	256012288.00	0.13
LLLPLDFR	4.27	0.02	256.04	1.33	1.65	0.11	1.01	0.02	3.24	0.09	1.45	0.12	75762439.00	0.15
LLLLPLDFR	4.49	0.02	269.16	1.38	1.26	0.06	1.36	0.10	2.45	0.12	1.64	0.09	51079561.00	0.17

B

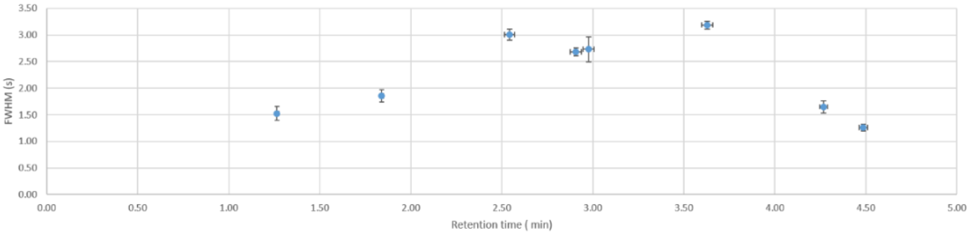

C

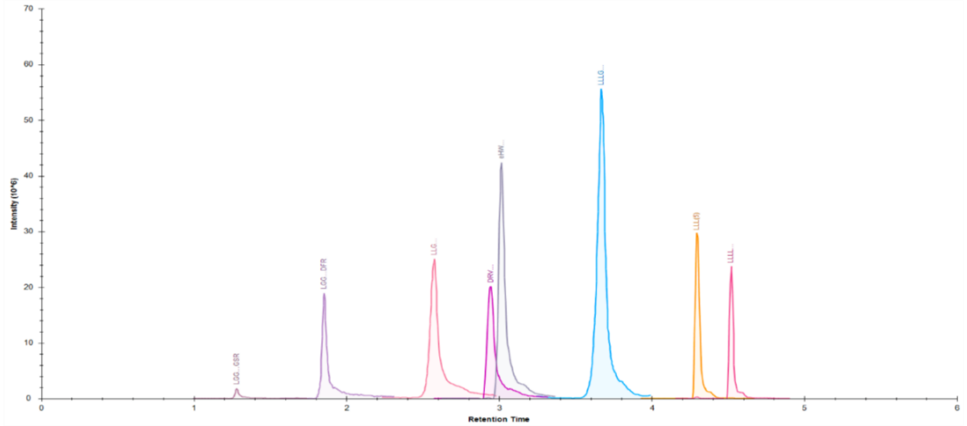

D

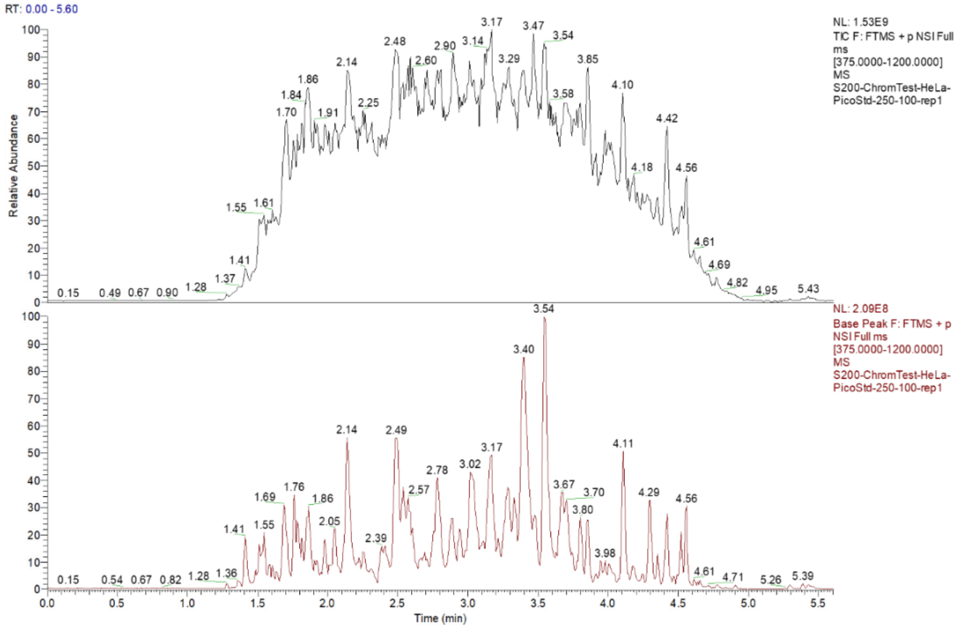

Supplemental Fig. S13: Chromatographic performance of the 200 samples per day method. *A,* Parameters describing the chromatographic performance for eight peptides of the PicoSure synthetic peptide standard. *B,* Retention time variation versus the variation in FWHM for quadruplicate measurements. *C,* Extracted chromatogram for the eight synthetic peptides. *D,* The upper panel shows the TIC and the lower panel the base peak chromatogram for the HeLa spiked in with the peptide standard.

A

Peptide	RT(min)	stdev	RT (sec)	stdev1	FWHM(s)	stdev2	TailFWHM	stdev3	Width13.4(s)	stdev4	Tail13.4	stdev5	Area	RSD
LGGGPGGGDGSR	0.95	0.01	56.97	0.61	1.29	0.06	1.14	0.26	2.88	0.64	1.52	0.57	600725.00	0.55
LGGGPGGGDFR	1.35	0.01	81.26	0.64	1.56	0.20	1.07	0.17	3.29	0.23	1.43	0.07	17288474.25	0.27
LLGGPGGDFR	1.72	0.00	103.09	0.21	1.87	0.13	1.13	0.27	4.27	0.31	1.48	0.10	34623171.50	0.16
DRVYIHPF	1.87	0.00	112.12	0.14	1.70	0.04	0.85	0.10	3.52	0.07	1.41	0.08	16230996.75	0.17
E[-18]HWSYGLRPG	1.90	0.00	113.82	0.15	1.72	0.06	0.90	0.10	3.30	0.20	1.27	0.11	19783967.00	0.15
LLLGPGDFR	2.14	0.01	128.26	0.44	1.80	0.10	1.21	0.18	3.90	0.06	1.11	0.05	79521735.50	0.12
LLLPLDFR	2.41	0.01	144.52	0.57	1.30	0.06	1.32	0.11	2.48	0.04	1.63	0.07	46662229.75	0.05
LLLLPLDFR	2.52	0.01	151.49	0.56	1.08	0.04	1.37	0.10	2.06	0.02	1.63	0.10	99111423.00	0.05

B

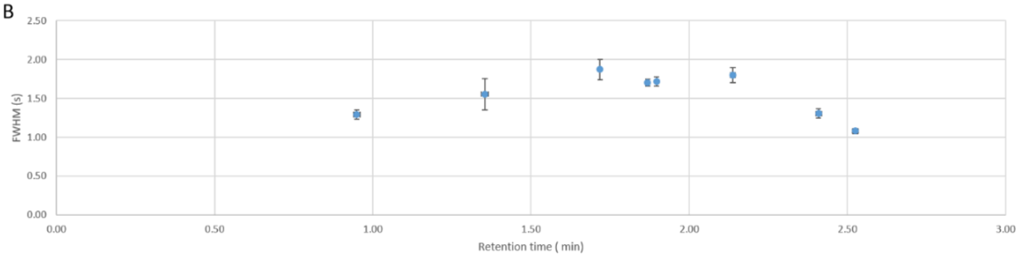

C

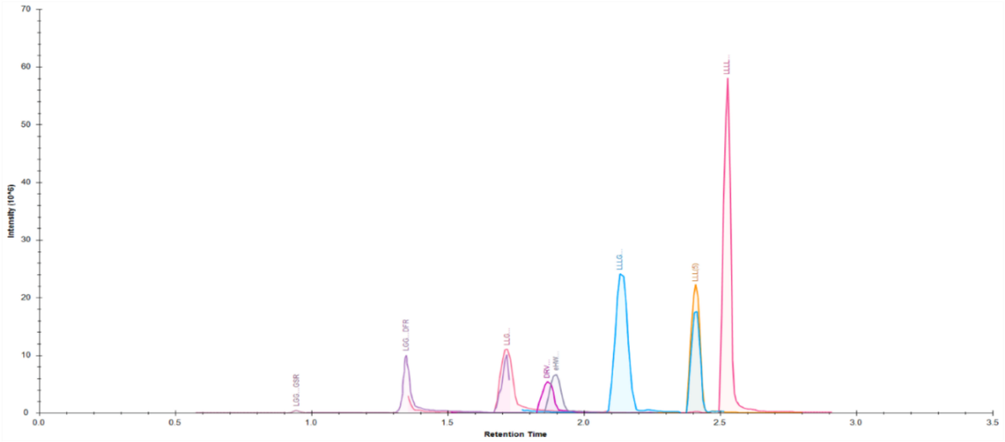

D

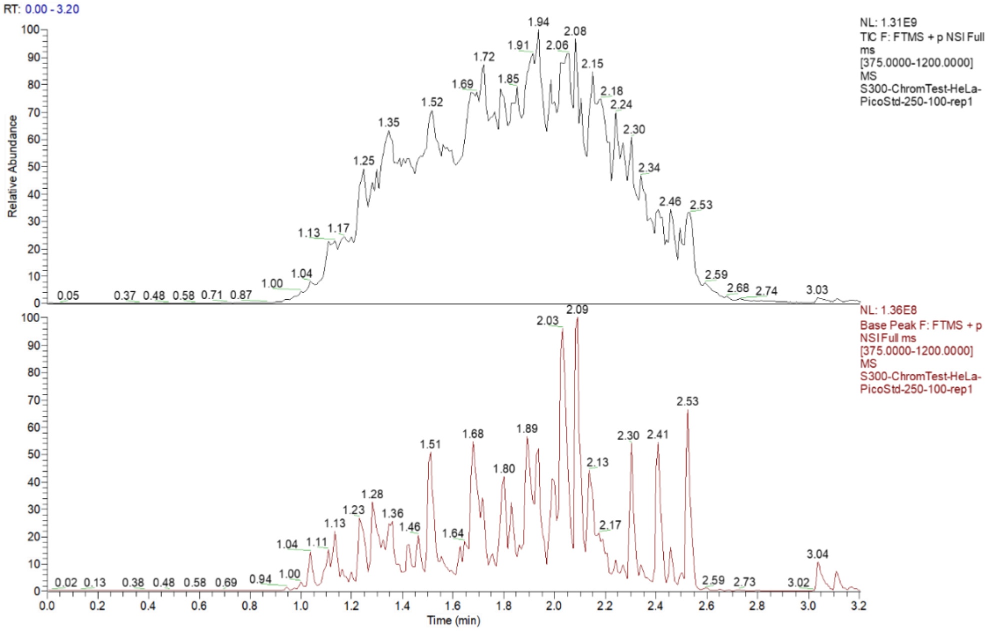

Supplemental Fig. S14: Chromatographic performance of the 300 samples per day method. *A,* Parameters describing the chromatographic performance for eight peptides of the PicoSure synthetic peptide standard. *B,* Retention time variation versus the variation in FWHM for quadruplicate measurements. *C,* Extracted chromatogram for the eight synthetic peptides. *D,* The upper panel shows the TIC and the lower panel the base peak chromatogram for the HeLa spiked in with the peptide standard.

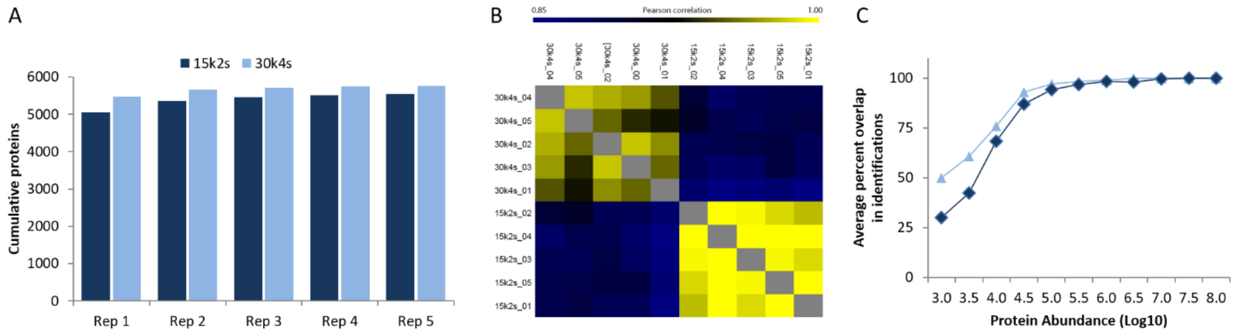

Supplemental Fig. S15: DIA method comparison on the Evosep One. *A,* Cumulative numbers of proteins for both methods. *B,* Heatmap of Pearson correlation coefficients, illustrating the reproducibility within and between the two methods. *C,* Overlap of identifications across the abundance rank for both DIA methods.
